# Exchanges of genomic domains between poliovirus and other cocirculating species C enteroviruses reveal a high degree of plasticity

**DOI:** 10.1038/srep38831

**Published:** 2016-12-13

**Authors:** Maël Bessaud, Marie-Line Joffret, Bruno Blondel, Francis Delpeyroux

**Affiliations:** 1 Institut Pasteur, Unité de biologie des virus entériques, Paris, France; 2 INSERM, U994, Paris, France

## Abstract

The attenuated Sabin strains contained in the oral poliomyelitis vaccine are genetically unstable, and their circulation in poorly immunized populations can lead to the emergence of pathogenic circulating vaccine-derived polioviruses (cVDPVs). The recombinant nature of most cVDPV genomes and the preferential presence of genomic sequences from certain cocirculating non-polio enteroviruses of species C (EV-Cs) raise questions about the permissiveness of genetic exchanges between EV-Cs and the phenotypic impact of such exchanges. We investigated whether functional constraints limited genetic exchanges between Sabin strains and other EV-Cs. We bypassed the natural recombination events by constructing 29 genomes containing a Sabin 2 capsid-encoding sequence and other sequences from Sabin 2 or from non-polio EV-Cs. Most genomes were functional. All recombinant viruses replicated similarly *in vitro*, but recombination modulated plaque size and temperature sensitivity. All viruses with a 5′UTR from Sabin 2 were attenuated in mice, whereas almost all viruses with a non-polio 5′UTR caused disease. These data highlight the striking conservation of functional compatibility between different genetic domains of cocirculating EV-Cs. This aspect is only one of the requirements for the generation of recombinant cVDPVs in natural conditions, but it may facilitate the generation of viable intertypic recombinants with diverse phenotypic features, including pathogenicity.

Recombination between viral RNA genomes is a well-known phenomenon in poliovirus (PV) and other enteroviruses (EVs). Intra- and intertypic recombination shapes the genetic diversity of these viruses, which often have mosaic genomes. Nevertheless, little is known about the permissiveness of intertypic genetic exchanges and their possible impact on phenotype.

PV is the etiological agent of paralytic poliomyelitis, a disease characterized by acute flaccid paralysis due to the destruction of motor neurons following PV replication^1^. The three serotypes of PV belong to *Enterovirus species C* (EV-C) (genus *Enterovirus*, family *Picornaviridae*). In addition to PV, the EV-C species includes more than 20 other serotypes defined according to the genomic sequence encoding their capsid proteins.

EVs are small non-enveloped viruses containing a single-stranded RNA genome of positive polarity of approximately 7.5 kb in length. This genome consists of two untranslated regions (5′UTR and 3′UTR) flanking a single large open reading frame. The 5′UTR and 3′UTR contain secondary structures involved in initiating the translation and replication of the genome^2^,^3^,^4^. The open reading frame is translated into a single polyprotein that is proteolytically processed to yield the four capsid proteins (VP1–4), which are encoded by the P1 region of the genome, and the non-structural proteins, encoded by the P2 and P3 regions. The non-structural proteins are involved in viral replication; they include proteases and the RNA-dependent RNA polymerase 3D.

The World Health Organization program for the global eradication of poliomyelitis, which was launched in 1988, has been largely successful. This program has mostly been based on vaccination with the oral polio vaccine (OPV), which is composed of live attenuated strains of the three PV serotypes, the Sabin 1, 2 and 3 strains. Most of the determinants of vaccine strain attenuation, impairing replication in the nervous system, are located in the 5′UTR and the region encoding the capsid proteins^5^. Many of these determinants also confer a temperature-sensitive (ts) phenotype on the strains^6^. The Sabin strains can replicate to high titers only in the digestive tract, conferring strong systemic and intestinal immunity, limiting subsequent PV replication and viral transmission in humans^7^. However, OPV strains are genetically unstable, and their circulation in poorly immunized populations can lead to genetic drift and the emergence of pathogenic circulating Sabin vaccine-derived PV (cVDPVs)^8^,^9^. More than 20 outbreaks of disease due to cVDPVs have been reported^10^,^11^,^12^. Most of the cVDPVs studied to date have recombinant genomes composed of mutated sequences from Sabin strains and sequences from non-polio EVs; with the non-polio EV sequences located within the regions P2, P3 or 3′UTR of the genome^8^,^13^,^14^,^15^,^16^, and in many cases within the 5′UTR^17^,^18^,^19^,^20^,^21^.

We previously analyzed the cVDPV strains implicated in two outbreaks of poliomyelitis in Madagascar in 2001–2002 (type 2 cVDPVs) and 2005 (type 2 and 3 cVDPVs)^15^,^17^,^22^. These cVDPVs have complex mosaic genomes that have emerged from a highly diverse EV-C ecosystem through recombination, mostly with coxsackievirus A (CV-A) sequences, often from type 13 (CV-A13) and CV-A17 viruses cocirculating in healthy children in the same area of the island. Some genomic sequences encoding non-structural proteins from several cVDPVs isolated in 2002 and 2005 display high levels of sequence identity to each other (>90%) and to a CV-A17 isolate collected in 2002^15^,^17^. The presence of closely related non-Sabin sequences in pathogenic cVDPVs collected three years apart suggested that the incorporation of particular non-polio EV-C sequences encoding non-structural proteins into cVDPVs may be favored. There may be several reasons for this, including preferred co-infection of the same host and cells by both parental strains, replication mechanisms favoring exchanges by intertypic recombination and the functional compatibility of the domains exchanged. The presence of these particular sequences may also result in recombinant cVDPVs with enhanced replication, spreading and/or neurovirulence.

Several studies have already investigated the genetic plasticity of PV *in vitro* and elucidated the role of non-polio EV-C sequences in the phenotype of recombinants. The strategy initially used involved cotransfecting or co-infecting cells and then studying the recombination events occurring between different viruses in permissive cells. Using such a strategy, we recently showed that a defective type 2 cVDPV genome with a small deletion at the 3′ end could be rescued by the cotransfection of cells with defective or infectious CV-A17 RNAs^23^. Many homologous or nonhomologous recombinants were obtained, with recombination hotspots in three regions encoding nonstructural proteins. Additional experiments showed that recombination within the 5′UTR of type 2 cVDPVs modified the replication properties of these viruses^24^.

Another strategy involved studying viruses generated from chimeric genomes constructed *in vitro*, with recombination sites at predetermined positions. By constructing recombinants between a type 2 cVDPV and its parental Sabin 2 strain, we were able to show that the EV-C sequences present in the 3′ half of the genome of this cVDPV contributed to its characteristics, including pathogenicity in transgenic mice expressing the PV cellular receptor gene (PVR-Tg21 mice)^25^. In another study, the 3′ half of a cVDPV was replaced with that of a Madagascan strain of CV-A17 cocirculating with this cVDPV. The recombinant was almost as neurovirulent as the cVDPV in transgenic mice^26^. These studies suggest that the incorporation of particular non-structural sequences from CV-A17 and CV-A13 into recombinant cVDPVs may be favored and that this may contribute to the emergence of pathogenic type 2 cVDPVs.

We investigated whether functional constraints limited the presence of particular CV-A sequences and favored the presence of others in recombinant cVDPVs, by evaluating the ability of the Sabin 2 genome to generate functional genomes through recombination with sequences from a wide range of cocirculating EV-Cs. Chimeric genomes were constructed from genomic sequences from isolates representative of the main EV-C lineages cocirculating in healthy children during the 2001–2002 poliomyelitis outbreak in Madagascar^15^,^22^. We assessed the potential roles of the genomic regions P2 and P3-3′UTR from non-polio EV-Cs, together with their respective 5′UTRs, in the growth properties and neurovirulence of recombinants carrying sequences encoding capsid proteins (P1 region) from the Sabin 2 strain.

We show here, for the first time, that the Sabin 2 genome can generate functional genomes through recombination with 5′UTR, P2 and P3-3′UTR domains from a wide range of cocirculating non-polio EV-Cs, without impeding primary replication mechanisms. However, recombination can modify phenotypic features, including pathogenicity.

## Materials and Methods

### Cell lines

HEp-2c cells were grown in Dulbecco’s modified Eagle’s medium (DMEM; PAA Laboratories, Pasching, Austria) supplemented with 5% (vol/vol) heat-inactivated newborn calf serum (NBCS; PAA Laboratories) and 2 mM L-glutamine (Invitrogen, Cergy-Pontoise, France), at 37 °C.

Human embryonic kidney 293 T/T7 cells, which constitutively express the T7 RNA polymerase, were kindly provided by Pierre Charneau (Institut Pasteur, Paris, France). They were grown in DMEM supplemented with 10% heat-inactivated fetal bovine serum (FBS; Gibco) and 2 mM L-glutamine, at 37 °C.

### Virus strains

CV-A11 isolate 66122, CV-A13 isolates 67001, 68095 and 68145, CV-A17 isolates 67610 and 68154 and EV-C99 isolate 68229 were obtained from stool samples collected in Madagascar in 2002^15^; their whole genomes were sequenced in a previous study^22^. PV-2 strain Sabin (referred to as Sabin 2) was recovered from pT7S2 (kindly provided by Andrew Macadam, NIBSC, Potters Bar), by transfecting 293 T/T7 cells (see below). PV-2 strain 4568-1/ISR98, which was used as a positive control for neurovirulence experiments, is a highly virulent cVDPV derived from the Sabin 2 strain that was isolated from sewage in Israel in 1998^27^.

All viruses were grown in HEp-2c monolayers in DMEM supplemented with 2% fetal calf serum and 2 mM L-glutamine, at 37 °C. Virus titers were evaluated in HEp-2c cells, by determining the number of 50% tissue culture infective dose units (TCID_50_) per ml according to the WHO standard protocol^28^.

All experiments with genetically engineered recombinant viruses were carried out in BSL-3 facilities.

### Generation of chimeric genomes and virus recovery

Chimeric genomes were produced by a previously described fusion PCR-based procedure^29^. The overlapping amplicons used to generate the complete chimeric genomes were generated by PCR, using viral sequences amplified by RT-PCR and inserted into plasmids as a matrix^29^. The primers are listed in Supplementary Tables S1 and S2 available online. The overlapping amplicons were purified, mixed and joined together by fusion PCR, as previously described^29^. The full-length DNA genomes were purified and used to transfect 293 T/T7 cells^29^. Transfected cells were incubated at 37 °C until full cell lysis (or for 7 days if no cell lysis was observed).

Transfected cell supernatants were collected, clarified by centrifugation, and 500 μL of the resulting viral suspension was used to infect 50–80% confluent HEp-2c cells grown in T-25 flasks. When cell lysis was complete, flasks were frozen and thawed three times. Cell supernatants were collected and used for a second passage in HEp-2c cells. The supernatant was clarified by centrifugation and the resulting viral suspension was stored at −80 °C until use. If no cell lysis was observed after the first passage, a second passage was performed. If no cytopathic effect was observed in the first seven days after infection, the supernatants of the first and second passages were clarified and subjected to a generic 5′UTR-targeting real-time RT-PCR in accordance with a previously described protocol^30^.

For all the recombinant viruses recovered, we investigated the recombination sites by sequencing with specific primers, with the BigDye Terminator v3.1 kit (Applied Biosystems, Courtabœuf, France) and an ABI Prism 3140 automated sequencer (Applied Biosystem).

### Growth kinetics

Growth kinetics assays were performed in 96-well culture plates seeded with 10^5^ HEp-2c cells per well and infected at a multiplicity of infection of 10^−2^ TCID_50_ per cell in DMEM supplemented with 2% (vol/vol) FBS and 2 mM L-glutamine. Plates were incubated at 37 °C, under a humidified atmosphere containing 95% air and 5% CO_2_, and they were frozen at 8, 24, 48 and 72 h post-infection (p.i.). Plates were subjected to three freeze-thaw cycles, and each supernatant was then collected and clarified by centrifugation. The viral titer of each supernatant (TCID_50_.mL^−1^) was evaluated on HEp-2c cells.

### Viral plaque assay

Cell monolayers in six-well culture plates (2 × 10^6 ^per well) were washed twice with DMEM without serum, and then infected with 400 μl per well of 10-fold serial dilutions of viral stock suspensions. Viral adsorption was allowed to proceed for 30 minutes, and the cells were then washed, covered with DMEM supplemented with 2% FBS, 2 mM L-glutamine and 1.2% Avicel RC-581 (FMC BioPolymers, Brussels, Belgium)^31^ and incubated at 37 °C under a humidified atmosphere containing 95% air and 5% CO_2_. After three days, the overlays were removed from the cells and the plaques were visualized by staining with crystal violet and their diameter was measured.

### Temperature sensitivity

The temperature sensitivity phenotype of viruses was evaluated by titrating the same virus stock at 37.0 °C (optimal temperature) and 40.2 °C (supraoptimal temperature), for five days. The reproductive capacity of viruses at supraoptimal temperature (RCT) was calculated as the difference between the log_10_ virus titers of the viral stock at 37.0 °C and 40.2 °C.

### Neurovirulence assay in PVR-Tg21 mice

All animal studies reported here were approved by and conducted in accordance with the guidelines of the Office of Laboratory Animal Care and the Institut Pasteur Ethics Committee. This study is registered under numbers 08188 (Experimental infection of mice with poliovirus) and CETEA 2013–0066. This work was performed in accordance with French and European regulations on the care and protection of the laboratory animals (EC Directive 2010/63, French Law 2013–118, February 6, 2013).

The neurovirulence of the viruses was assessed in homozygous PVR-Tg21 mice, which constitutively express the human PV receptor CD155 (generously provided by Akio Nomoto, University of Tokyo, Japan)^32^. Survival curves were generated by inoculating groups of six-week-old mice (equal numbers of males and females) intracerebrally (IC) with 40 μL of a viral suspension adjusted to 10^7.5^ TCID_50_/mL in DMEM supplemented with 2% (vol/vol) FBS, 100 units.mL^−1^ penicillin and 0.1 mg.mL^−1^ streptomycin (Sigma-Aldrich, Lyons, France).

We determined the viral dose (log_10_ TCID_50_) causing paralysis in 50% of the mice, the 50% paralytogenic dose (PD_50_), by inoculating groups of seven mice IC with 30 μL of a viral suspension of 10^5.3^ to 10^7.7^ TCID_50_.mL^−1^. Before IC inoculation, mice were anesthetized by the intraperitoneal injection of 0.25 mg xylazine (Rompun, Bayer, Leverkusen, Germany) and 2.5 mg ketamine (Imalgene, Merial, Lyons, France) in a total volume of 100 μL PBS. The animals were examined daily for 21 days post-inoculation, and paralysis and/or death were recorded. Survival curves were generated by the Kaplan-Meier method^33^, in R software^34^. The PD_50_ was calculated by the Reed and Muench method^35^.

Viruses were recovered by culturing cells from the central nervous system, so that their attenuation markers could be checked by sequencing. Briefly, dead or paralyzed mice were dissected in aseptic conditions and the spinal cord was collected and crushed in DMEM supplemented with 2% (vol/vol) FBS, 2 mM L-glutamine, 100 units.mL^−1^ penicillin and 0.1 mg.mL^−1^ streptomycin. Homogenates were centrifuged and the supernatants were used to inoculate subconfluent HEp-2c cells in T-25 flasks.

### Statistical analysis

Statistical analyses were conducted in R^34^. Survival curves were compared in log-rank tests carried out with the “survival” package^36^.

## Results

### Ability of the various Sabin 2/non-polio EV-C chimeric genomes to produce viable viruses

We first assessed whether non-capsid sequences from a large panel of circulating non-polio EV-Cs could give rise to functional recombinant Sabin 2 genomes. We selected a panel of representative non-polio EV-Cs isolated in Madagascar in 2001–2002 from healthy children living in the area in which the poliomyelitis outbreak occurred: three CV-A13 (67001, 68095 and 68145), two CV-A17 (67610 and 68154), one CV-A11 (66122) and one EV-C99 (68229). Two of these isolates (CV-A13 (68095) and CV-A17 (68154)) displayed high levels of sequence identity (>90%) to cVDPV strains isolated in 2002 and/or 2005, for various non-structural genomic regions^17^,^22^.

**Figure 1:**
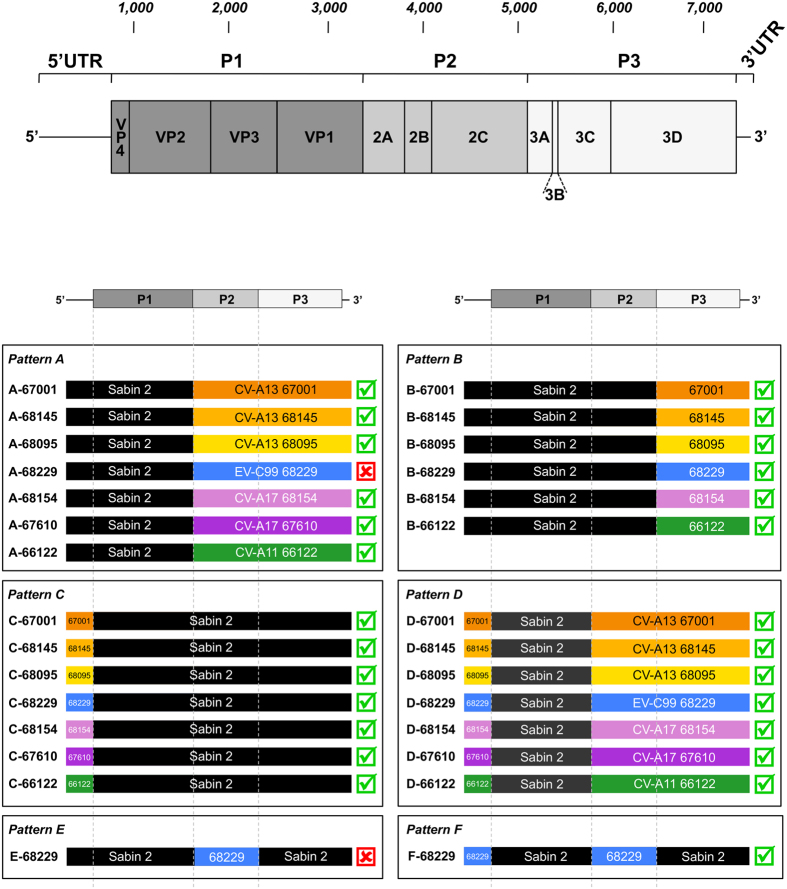
Viability of recombinant viruses with genomic patterns A to F. The genomic organization of the enterovirus genome is shown at the top. The name of the recombinant viruses is indicated on the left-hand side: the letter indicates the genomic pattern and the number indicates the non-polio enterovirus strain used to construct the chimeric genome. On the right-hand side, a green tick indicates the viability of the virus recovered after the transfection of HEp-2c cells with the recombinant genome, whereas a red cross indicates that no viable virus was obtained from the recombinant genome under the same conditions, even after two blind passages.

We used a previously developed fusion PCR-based procedure to engineer Sabin 2-derived chimeric genomes with recombination sites at precise nucleotide positions^29^. We constructed a set of chimeric Sabin 2/non-polio EV-C genomes with a Sabin 2 capsid-encoding sequence in common; the other sequences were from Sabin 2 or from non-polio EV-Cs (Fig. 1).

The genomic composition of the recombinants was used to investigate the respective roles of the various fragments from non-polio EV-C swapped with the corresponding regions of the Sabin 2 genome: the P2 and the P3-3′UTR fragments (pattern A), the P3-3′UTR fragment only (pattern B), the 5′UTR only (pattern C) and the 5′UTR together with the corresponding P2 and P3-3′UTR fragments (pattern D) (Fig. 1). Recombinants were named according to their pattern (A to D) followed by the name of the non-polio EV-C isolates selected for construction of the recombinant.

All but one (A-68229) of the genomes of patterns A to D gave rise to viable viruses causing the complete lysis of HEp-2c cell monolayers (Fig. 1). The A-68229 genome, consisting of the 5′UTR and the capsid-encoding region from Sabin 2, with the rest of its sequences (P2-P3-3′UTR) from EV-C99 68229, did not induce any cytopathic effect in HEp-2c cells even after two blind passages. Furthermore, real-time RT-PCR did not detect viral RNA in any of the cell supernatants. These results suggest an incompatibility between the P2 region of EV-C99 68229 and the 5′UTR of Sabin 2. We tested this hypothesis by constructing Sabin 2 recombinant genomes with only the P2 region of EV-C99 68229 (E-68229) or with both the 5′UTR and the P2 region of EV-C99 68229 (F-68229) (Fig. 1). As expected, only the latter gave rise to viable viruses. However, the incompatibility between the 5′UTR of Sabin 2 and the P2 region of EV-C99 68229 did not seem to be reciprocal, because the C-68229 genome, containing the 5′UTR from EV-C99 68229 and the P2 region from Sabin 2, was functional.

These results indicate that most of the P2 and P3-3′UTR sequences from circulating non-polio EV-C viruses tested here could generate functional viruses through recombination with Sabin 2 genomes. Furthermore, with only one exception, exchanges of P2 and P3-3′UTR sequences from these non-polio EV-C could be performed independently of their respective 5′UTRs.

### Phenotypic properties of recombinant viruses with genomic patterns A-D in cell cultures

We investigated whether the genomic sequences of non-polio EV-Cs affected the properties of the Sabin 2-derived recombinant viruses, by assessing three phenotypic characteristics in HEp-2c cells for Sabin 2 and each of the viable viruses displaying genomic patterns A to D: replication kinetics, plaque size in semi-solid medium and temperature sensitivity phenotype.

**Figure 2:**
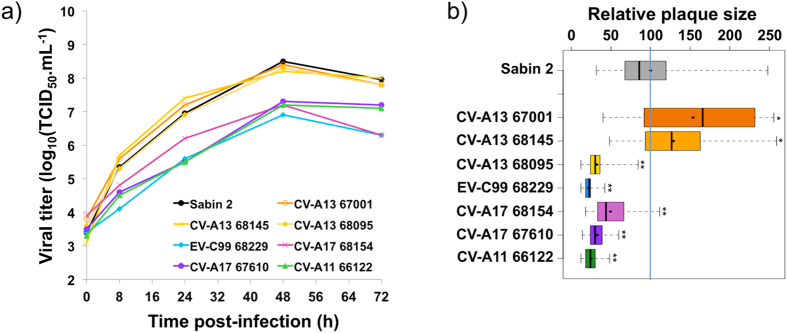
Replication kinetics and relative plaque sizes of the parental strains. (**a**) Replication kinetics curves. (**b**) Relative plaque size was calculated by taking the mean Sabin 2 plaque diameter as the reference (value of 100, indicated by the blue vertical line). For a given virus, the boxplot shows the first and third quartiles (which delimit the box), the median (thick bar) and the minimum and maximum values (thin bars); the mean is indicated by a diamond (♦). For each virus, mean relative plaque size was compared with that of Sabin 2 in the Welsh *t* test for datasets with unequal variances; *p*-values are indicated as * < 0.03, ** < 10^−5^.

**Figure 3:**
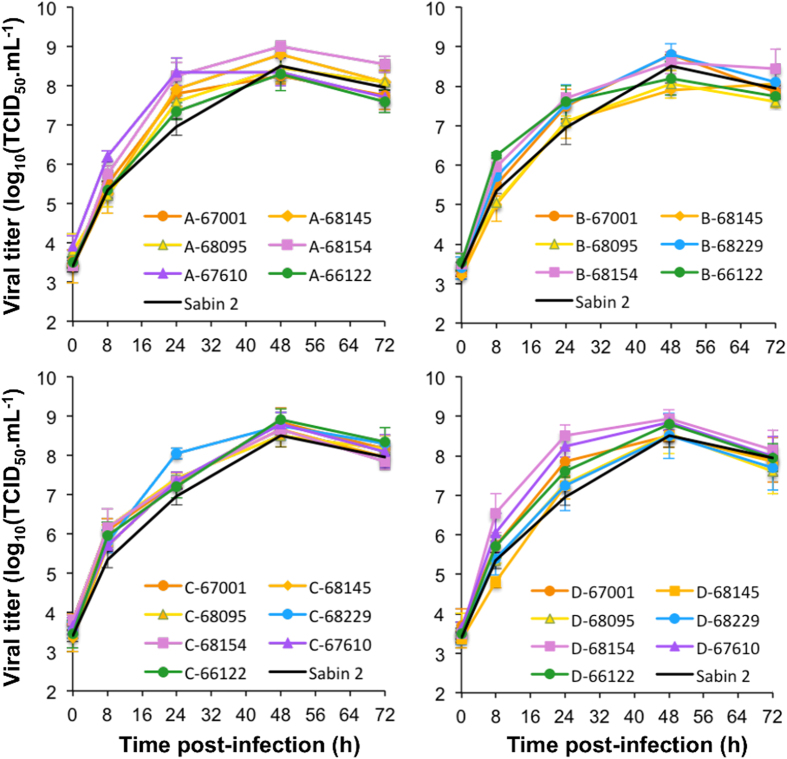
Replication kinetics assays. We compared replication kinetics between the recombinant viruses with genomic patterns A-D and Sabin 2 (black curves) in HEp-2c cells at 37 °C. Error bars indicate the standard deviations calculated in two experiments.

We first compared the growth kinetics in HEp-2c cells of Sabin 2, the other parental viruses and recombinants displaying genomic patterns A to D. Sabin 2 and the three CV-A13 led to the complete lysis of HEp-2c cells and reached a titer >10^8^ TCID_50_.mL^−1^ 48 h post infection, whereas the other parental strains only partially destroyed the monolayer and reached lower titers (Fig. 2a). All the recombinant viruses of panels A-D led to the full lysis of the cell monolayers and recombination had no major deleterious effects on viral growth *in vitro*, regardless of recombination pattern and parental sequences. However, slight differences could be seen at 24 hours post-infection (Fig. 3).

**Figure 4:**
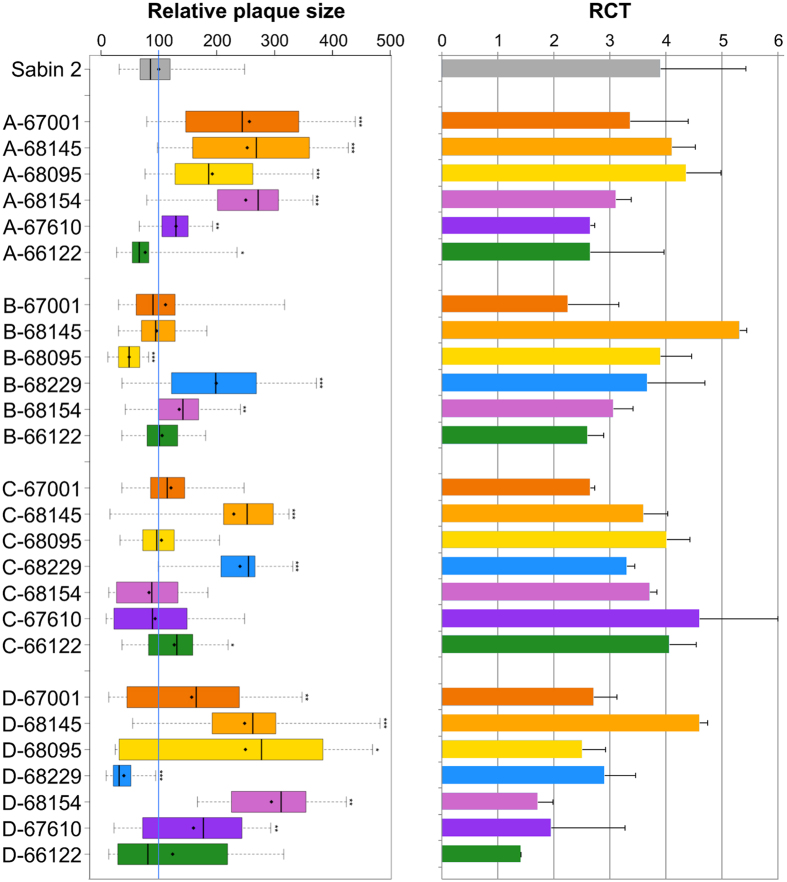
Comparison of plaque size and RCT values between the viruses of sets A, B, C and D and Sabin 2. Relative plaque size was calculated, taking mean Sabin 2 plaque diameter as the reference (value of 100, indicated by the blue vertical line). For a given virus, the boxplot shows the first and third quartiles (delimiting the box), the median (thick bar) and the minimum and maximum values (thin bars); the mean is indicated by a diamond (♦). For each virus, mean relative plaque size was compared with the mean plaque size for Sabin 2, in a two-sided Welsh *t* test for unequal variances; *p*-values are indicated if lower than 0.05 (* < 0.05, ** < 0.01, *** < 0.001). The temperature sensitivity of the viruses was evaluated by titrating virus stocks at 37 °C and 40.2 °C and calculating reproductive capacity at supraoptimal temperature (RCT) as indicated in the Materials and Methods. The mean of two experiments is shown, with error bars indicating the standard deviations.

Taking Sabin 2 plaque size as the reference (value set to 100), we found that the CV-A13 67001 and 68145 isolates gave plaques significantly larger than Sabin 2, whereas the other parental strains gave tiny plaques (Fig. 2b). The recombinant viruses with genomic patterns A, B, C and D had relative mean plaque sizes of 39.7 (D-68229) to 249.5 (D-68154) (Fig. 4). Most (17/28) formed plaques significantly larger than those formed by Sabin 2, but a few (3/28) formed smaller plaques. Plaque phenotype appeared to have been modified for nine of the 12 viruses with genomic patterns A and B, which differed from Sabin 2 in terms of their P2 and/or P3-3′UTR regions, and for three of the seven viruses with pattern C, which differed from Sabin 2 only in terms of their 5′UTR. Thus, recombination events within the 5′UTR or P2-P3-3′UTR sequences appeared to modify the plaque phenotype of Sabin 2-derived recombinant viruses, by increasing or decreasing plaque size.

Finally, the temperature sensitivity phenotype of the viruses was evaluated by calculating RCT values, *i*.*e.* the difference in titer (log_10_) between virus stocks at 37.0 °C and 40.2 °C. We found that Sabin 2 had an RCT value of 3.9 ±1.5 (Fig. 4). The three CV-A13 isolates were heat-resistant (RCT values <1) whereas the growth of the other non-polio parental strains was impaired at high temperature (RCT values >2). The RCT values of the recombinant viruses ranged from 1.4 ± 0.1 (D-66122) to 5.3 ± 1.2 (B-68145). These results indicate that intertypic recombination in non-capsid sequences can modify the temperature sensitivity phenotype. Nevertheless, none of the recombinant viruses had RCT values ≤1.0, by contrast to most cVDPVs, which display no growth impairment at high temperatures^17^,^26^.

Overall, these results show that the genetic exchanges performed in these viable recombinants had no significant effect on their overall ability to replicate in cells, but they did result in a diversity of plaque sizes and features of the ts phenotype. No correlations were observed between RCT value and plaque size or between one of these phenotypic features and certain recombination patterns or EV-C domains. Furthermore, no correlations were observed between the phenotypic characteristics of the non-polio parental strains and those of the corresponding recombinant viruses. In particular, the phenotypic properties of the viruses of panel D (the genomes of which consisted mainly of sequences from non-Sabin 2 viruses) did not appear to be linked to those of their respective non-polio parental strains.

### Neurovirulence in *PVR-Tg21 mice* of the recombinant viruses with genomic patterns A-D

**Figure 5:**
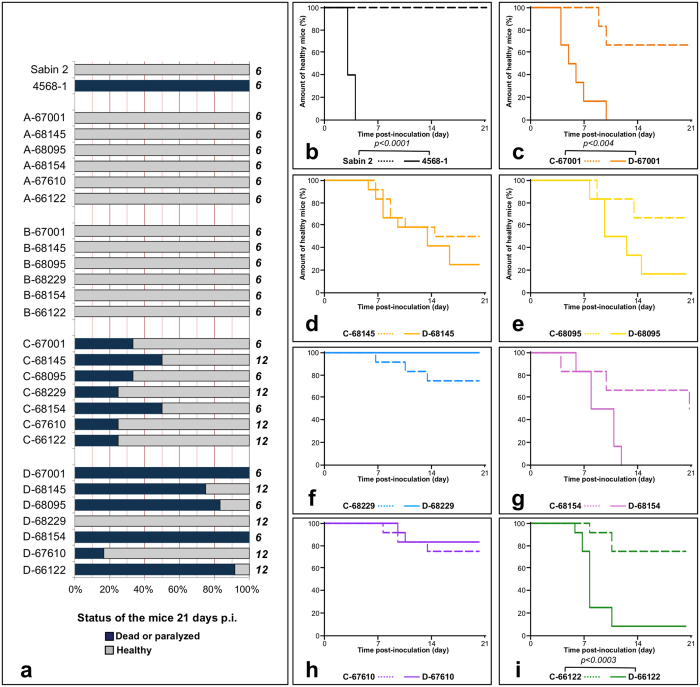
Neurovirulence in transgenic mice. Mice were inoculated intracerebrally with the viruses. (**a**) Proportion of dead or paralyzed mice 21 days after inoculation. On the right of each bar, the number of mice inoculated is indicated. (**b**) Survival curves obtained for Sabin 2 and ISR4568-1, a highly neurovirulent cVDPV used as a positive control. (**c-i**) Survival curves obtained for viruses with genomic patterns C and D. The survival curves were compared in log-rank tests; *p*-values are indicated if < 0.05.

We assessed the neurovirulence of the recombinant viruses in homozygous transgenic PVR-Tg21 mice, which express the PV receptor and are susceptible to PV infection. The animals were examined daily for 21 days post-inoculation, and paralysis and death were recorded. As expected, no symptoms were detected in these mice after inoculation with the attenuated Sabin 2 strain, whereas the Sabin 2-derived VDPV strain 4568-1/ISR98, used as a positive control, killed 100% of the mice within 4 days (Fig. 5a and b).

All recombinant viruses with genomic patterns A and B had an attenuated phenotype, as no paralyzed or dead mice were observed among the six mice inoculated with these viruses (Fig. 5a). Thus, substitutions in the P2 and/or P3/3′UTR sequences of the Sabin 2 genome were not sufficient to confer a neurovirulent phenotype on the recombinants.

By contrast, all viruses with genomic pattern C were able to induce paralysis in mice, as 25 to 50% of the mice inoculated with each of these viruses developed paralysis (Fig. 5a). Thus, the 5′UTR from each of the non-polio EV-Cs decreased the attenuation of the Sabin 2 recombinants.

All but one (D-68229) of the viruses with genomic pattern D also caused paralysis in 16 to 100% of mice, depending on the recombinant concerned (Fig. 5a). A comparison of survival curves (Fig. 5b–i) indicated that two of these viruses, D-66122 and D-67001, were significantly less attenuated than their corresponding homologs with genomic pattern C (*p* < 0.001 and 0.0003, respectively, Fig. 5c and i).

**Figure 6:**
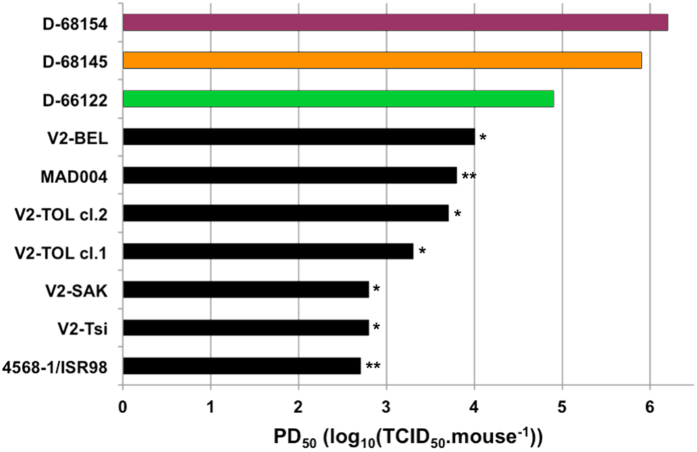
Doses of virus inducing the paralysis or death of 50% of the mice (PD_50_). For D-68154, D-68145 and D-66122, three groups of seven PVR-Tg21 mice were inoculated intracerebrally with a single dose of virus (ranging from 10^3.8^ to 10^6.3^ TCID50 per mouse). The PD50 of these three viruses were compared with those of cVDPVs already determined in previous works (* ref. 17 and ** ref. 26).

Finally, we compared the neurovirulence of the viruses engineered in this study with that of natural cVDPVs, by determining the PD_50_ (i.e. the logarithm of the viral dose inducing paralysis or death in 50% of the mice) of three viruses with genomic pattern D: D-68154, D-68145 and D-66122. These three viruses had non-polio EV sequences from CV-A17, CV-A13 and CV-A11 isolates, respectively (Fig. 6). These engineered recombinant viruses had PD_50_ values of 6.1, 5.9 and 4.8, respectively, indicating more moderate neurovirulence than for the type 2 cVDPVs isolated in Madagascar, which had PD_50_ values of 2.7 to 4.0 (Fig. 6)^17^,^26^.

In addition to the attenuation determinant located within the 5′UTR (the adenine residue in position 481), the attenuated phenotype of Sabin 2 is known to result at least in part from a determinant at nucleotide 2909, corresponding to an isoleucine residue in position 143 of the VP1 capsid protein^37^,^38^. We investigated whether the loss of attenuation of the set of recombinants with genomic patterns C and D was associated with a nucleotide mutation affecting this codon, by recovering a sample of each of the recombinant viruses from one dead or paralyzed mouse and sequencing the VP1-encoding regions of their genomes. All the viruses recovered from dead or paralyzed mice had an unmodified isoleucine residue in position 143 of VP1, demonstrating a lack of direct association between the neurovirulent phenotype and a mutation affecting the corresponding codon. Furthermore, we investigated whether mutations were acquired during the multiplication of recombinants in mice, by fully sequencing the genomes of three viruses (pattern D) recovered from dead or paralyzed mice. Relative to the sequences of the corresponding recombinants before inoculation, a few mutations were observed in the genome, but most were synonymous (see Supplementary Fig. S1 online). Rare missense mutations were found in the genomic regions encoding the viral capsid or nonstructural proteins but no two such mutations were found in the same codon or affecting the same protein. It is therefore difficult to determine whether they played a role in the capacity of the recombinant to replicate in mice or not.

These results confirmed the key role of the 5′UTR sequence in the neurovirulent phenotype of some cVDPVs and showed that other genomic regions could also modulate this phenotype.

## Discussion

The repeated isolation of pathogenic cVDPVs containing closely related non-polio EV-C genomic sequences suggested that particular non-polio EV-C sequences might be selected in viable recombinant genomes and that this might favor the emergence of type 2 cVDPVs. Indeed, genomic sequences closely related to those of Madagascan CV-A17 and CV-A13 strains were identified in various lineages of cVDPVs that emerged in 2002 and 2005 on the island^15^,^17^,^22^. The emergence of these recombinant cVDPVs in a population in which other EV-Cs were circulating raises questions about the permissiveness of intertypic genetic exchanges and the possible impact of these exchanges on phenotype. Recombination between different enteroviruses may be restricted by many bottlenecks, such as the frequency with which the host and cells are co-infected with these two viruses or the frequency of recombination events, which may depend on genomic differences and replication processes. Functional incompatibilities between the exchanged genomic domains and/or the encoded proteins may also prevent the emergence of new viruses through recombination. In this study, we focused on the functional incompatibilities that might have prevented the emergence of pathogenic cVDPVs through recombination events between Sabin 2 and non-polio EV-Cs circulating in Madagascar during the 2001–2002 outbreak. We generated the recombinant genomes used here *in vitro,* and used them to transfect permissive cells, thereby bypassing the other natural recombination processes and mechanisms mentioned above. To focus on functional incompatibilities, we thus arbitrarily assumed that each pair of viruses had similar co-infection capacities and recombination frequencies.

The molecular method used to construct the recombinant genomes made it possible to obtain several sets of recombinant viruses in which Sabin 2 sequences and sequences from field non-polio EV-Cs were combined, with precisely located breakpoints at functional junctions. The non-polio EV-Cs selected for this study were highly relevant, as they were circulating in Madagascar when the 2001–2002 outbreak occurred. Four sets of genomes were thus generated (patterns A to D) to decipher the respective importance of the 5′UTR, P2 and P3-3′UTR regions in the recombinant virus phenotype. All the viruses in each set displayed identical patterns of genomic recombination, facilitating comparative studies.

Surprisingly, we found that Sabin 2 sequences were highly compatible with homologous non-polio sequences from other EV-Cs, because only one of the 27 genomes with patterns A to D tested was not viable. No deleterious effects on replication *in vitro* were observed for the viable viruses generated by recombination between Sabin 2 and other EV-Cs, regardless of the recombination site, because all the viable viruses in sets A to D displayed growth kinetics similar to that of Sabin 2 in HEp-2c cells. These results indicate that, in many EV-Cs, the various genomic domains have remained functionally compatible with those of Sabin 2 throughout evolution and differentiation. Recombination events have probably helped to maintain this compatibility, by conferring advantages on the recombinant viruses. It has recently been shown that intratypic recombination enhances adaptability, viral spread and the virulence of PV-1^39^. Intertypic recombination probably acts in a similar manner, modulating genetic drift to maintain functional compatibility between genomic domains from different viruses. Among EV-Cs, PV genomes appear to be particularly likely to generate viable PV capsid-harboring viruses through recombination with other EV-Cs, whereas the reciprocal phenomenon (i.e. the generation of genomes with a non-polio capsid-encoding sequence associated with other sequences from PVs) seems to lead mostly to impaired or defective viruses^40^.

Despite the high level of compatibility, there are some restrictions, as revealed by the non-viability of two genomes, A-68229 and E-68229. Our results suggest that the Sabin 2 5′UTR is incompatible with the P2 region of EV-C 99–68229. This incompatibility may be due to intra- or intermolecular interactions during replication, because the 2 C region contains the *cis*-acting replication element (*cre*), a 61 nt-long sequence involved in initiating the synthesis of negative and positive genomic strands through VPg uridylation^2^. However, the 26 nt-long minimal *cre* sequences present in the genomes of the two viruses are identical (see Supplementary Fig. S2).

Alternatively, this incompatibility may be due to a requirement for specific interactions between the 5′UTR and the membrane-bound 2 C protein^41^, which has several key functions in the viral replication cycle. This protein is part of the replication complex, in which it acts as an NTPase, and it is also involved in viral morphogenesis^2^,^42^,^43^. It contains RNA-binding domains but, to our knowledge, no direct interactions have been observed between this protein and the 5′UTR.

Our previous molecular analysis of the EV-Cs sampled in Madagascar in 2002 identified only one EV-C99 lineage, comprising several isolates, including 68229, suggesting that members of this lineage did not readily recombine with other EV-Cs in this epidemiological context^15^,^22^. These previous analyses and the data presented here suggest that there may be rare functional barriers limiting the emergence of specific recombinants within the EV-C species. Nevertheless, in our model, these barriers were easily overcome by additional recombination events.

The viable recombinants constructed in this work had similar replication rates in cell culture, but clearly different phenotypes. They displayed a wide range of plaque sizes and temperature sensitivity phenotypes, these two phenotypic markers being widely used historically to characterize EVs and to differentiate between vaccine strains and wild PV isolates^44^. The ts phenotype is partly correlated with the attenuation of PV strains^45^, but the molecular mechanisms affecting this phenotype and the plaque size of a given virus are complex, rendering these phenotypes difficult to interpret^46^. Nevertheless, this considerable phenotypic heterogeneity reveals the tremendous impact of recombination on the mechanisms underlying phenotype. Recombination generates highly diversified panels of EV-Cs, from which those best adapted to the local context can emerge and spread.

The non-polio EV sequences found in the P2-P3 regions of pathogenic cVDPVs isolated in Madagascar were mostly related to cocirculating CV-A13 and CV-A17 viruses^15^,^17^,^22^. This suggests that nonstructural genomic sequences from CV-A13 and CV-A17 may be selected in viable recombinant viruses with a Sabin 2 capsid and could contribute to particular gains of fitness or virulence. However, viable type 2 recombinant viruses with similar replicative properties were obtained during this study, through recombination events between Sabin 2 and non-polio sequences from all the EV-C types studied, and no particular properties were associated with CV-A13 or CV-A17 sequences. The presence of closely related non-polio EV-C sequences together in various cVDPV lineages found in Madagascar is therefore probably not due to the intrinsic ability of these sequences to generate pathogenic cVDPVs. Instead, it is more likely to result from the advantages these sequences conferred on these lineages in the Madagascan epidemiological context. The recombination events and the subsequent selection process are driven by many factors, and this may explain why some genomic sequences appeared to be more likely than others to recombine with each other. For example, the two parental strains must be able to infect the same cell type for recombination to occur. The mechanism of recombination is also constrained by complex rules that are currently poorly understood and that presumably prevent recombination from occurring between some sequences or in certain regions of the genome.

Previous studies have shown that the PV/non-PV breakpoints in the genomes of recombinant cVDPVs are preferentially located within the P2 region^15^,^17^, at the end of the 2A- or 2B-encoding region, although they may also be found close to the P1-P2 junction in some cases. Given the tremendous diversity of these sites in natural recombinants, we arbitrarily chose to create recombination sites at the VP1-2A (P1-P2) and 2C-3A (P2-P3) junctions. We cannot exclude the possibility that this use of fixed sites of recombination may have introduced differences in the permissiveness and characteristics of the constructed recombinants relative to natural recombinants with other recombination sites. However, the construction and characterization of two Sabin 2/CA17 recombinants with recombinant junctions at the VP1-2A and at the end of the 2 A region did not lead to significant phenotypic differences in a previous study^25^.

As indicated by previous studies^25^,^26^, the 5′-half of the genome of cVDPVs plays a key role in the pathogenicity of type 2 cVDPVs. In this study, none of the viruses of genomic patterns A and B (which have an unmodified Sabin 2 5′UTR sequence) was neurovirulent, whereas almost all the viruses of genomic patterns C and D caused disease in mice, confirming the crucial importance of the 5′UTR in the loss of attenuation of Sabin 2-derived recombinants. These results are consistent with observations that the 5′UTR of pathogenic type 2 cVDPVs is modified by mutations affecting the adenine nucleotide in position 481, which is known to be a major determinant of attenuation of Sabin 2, or by recombination with other EV-Cs^15^,^17^,^18^,^19^,^21^,^37^,^47^,^48^,^49^,^50^. The key role of the 5′UTR in the pathogenicity of PVs has also been demonstrated for serotypes 1 and 3^38^,^51^,^52^,^53^. The finding that most of the 5′UTRs from the cocirculating EV-C could contribute to the reversion of the attenuated Sabin 2 strain to a neurovirulent phenotype remains particularly surprising. It indicates that these 5′UTR domains have retained the ability to promote replication in neuronal cells throughout evolution and differentiation processes.

However, some viruses with identical 5′UTR sequences displayed different levels of attenuation, indicating a possible effect of the 3′ half of the genome on pathogenicity. The contribution of the 3′ half of the genome to the pathogenicity of PVs was demonstrated in previous studies^25^,^26^,^54^,^55^, but the precise underlying mechanisms remain to be deciphered. These mechanisms may involve different pathways, including, in particular, the replication machinery, which mobilizes proteins encoded by the non-structural regions of the genome and three-dimensional RNA structures within the 3′UTR^56^. Furthermore, substitutions in the amino-acid sequence of the 3D polymerase can modulate diversity among quasispecies^57^,^58^,^59^. The loss of quasispecies diversity has been correlated with attenuation for PV^57^,^58^,^59^ and various plus-stranded RNA viruses, such as the yellow fever 17D vaccine strain^60^.

The moderately neurovirulent phenotype of the viruses of panels C and D may also be due to the absence of specific mutations within the Sabin 2-derived regions of their genomes. Such mutations are probably necessary for the increase in fitness required for field cVDPVs to emerge as pathogenic strains. In particular, residue 143 of VP1, which is known to be a determinant of attenuation^37^, is generally found to be mutated in cVDPVs isolated from patients^15^,^17^,^18^,^19^,^21^,^49^,^50^. This result is consistent with the finding that most pathogenic cVDPVs circulate for months before causing poliomyelitis cases^8^,^10^. This period of circulation may be required for the virus to accumulate mutations that increase its fitness and, thus, its neurovirulence.

In conclusion, using recombinants between Sabin 2 and various non-polio EV-Cs generated *in vitro*, we have demonstrated striking genetic and functional compatibility between different genetic domains, including the 5′UTR, and the P2 and the P3-3′UTR domains, facilitating the efficient replication of most intertypic recombinants in cell culture. This compatibility suggests that viral functions and interactions are well conserved within the EV-C species. However, other parameters potentially favoring certain types of recombinants over others, such as the capacity of parental viruses to coinfect the same host and cells, the frequency of recombination and the selection of preferential recombination sites, remain to be evaluated. The results reported here indicate that a high degree of functional compatibility between the genetic domains of various cocirculating EV-Cs may favor intertypic recombination, generating viruses with highly diversified phenotypic features, including neurovirulence.

## Additional Information

**How to cite this article**: Bessaud, M. *et al*. Exchanges of genomic domains between poliovirus and other cocirculating species C enteroviruses reveal a high degree of plasticity. *Sci. Rep.* **6**, 38831; doi: 10.1038/srep38831 (2016).

**Publisher's note:** Springer Nature remains neutral with regard to jurisdictional claims in published maps and institutional affiliations.

## Supplementary Material

Supplementary Information
