# Supplementary material for: Exchanges of genomic domains between poliovirus and other cocirculating species C enteroviruses reveal a high degree of plasticity
Source: Sci Rep. 2016 Dec 13;6:38831. doi: 10.1038/srep38831 (PMC5153852; doi:10.1038/srep38831)
Supplement: Supplementary Information [file srep38831-s1.pdf]

Exchanges of genomic domains between poliovirus and other cocirculating species C enteroviruses reveal a high degree of plasticity

Maël Bessaud, Marie-Line Joffret, Bruno Blondel, Francis Delpeyroux

Supplementary Information

Supplementary Fig. S1. Nucleotide divergences between three viruses recovered from dead or paralized mice and the corresponding viruses before inoculation. The consensus sequences were determined through Sanger sequencing. The non-synonymous divergences are in red. The numbering is according to that of the parental strains.

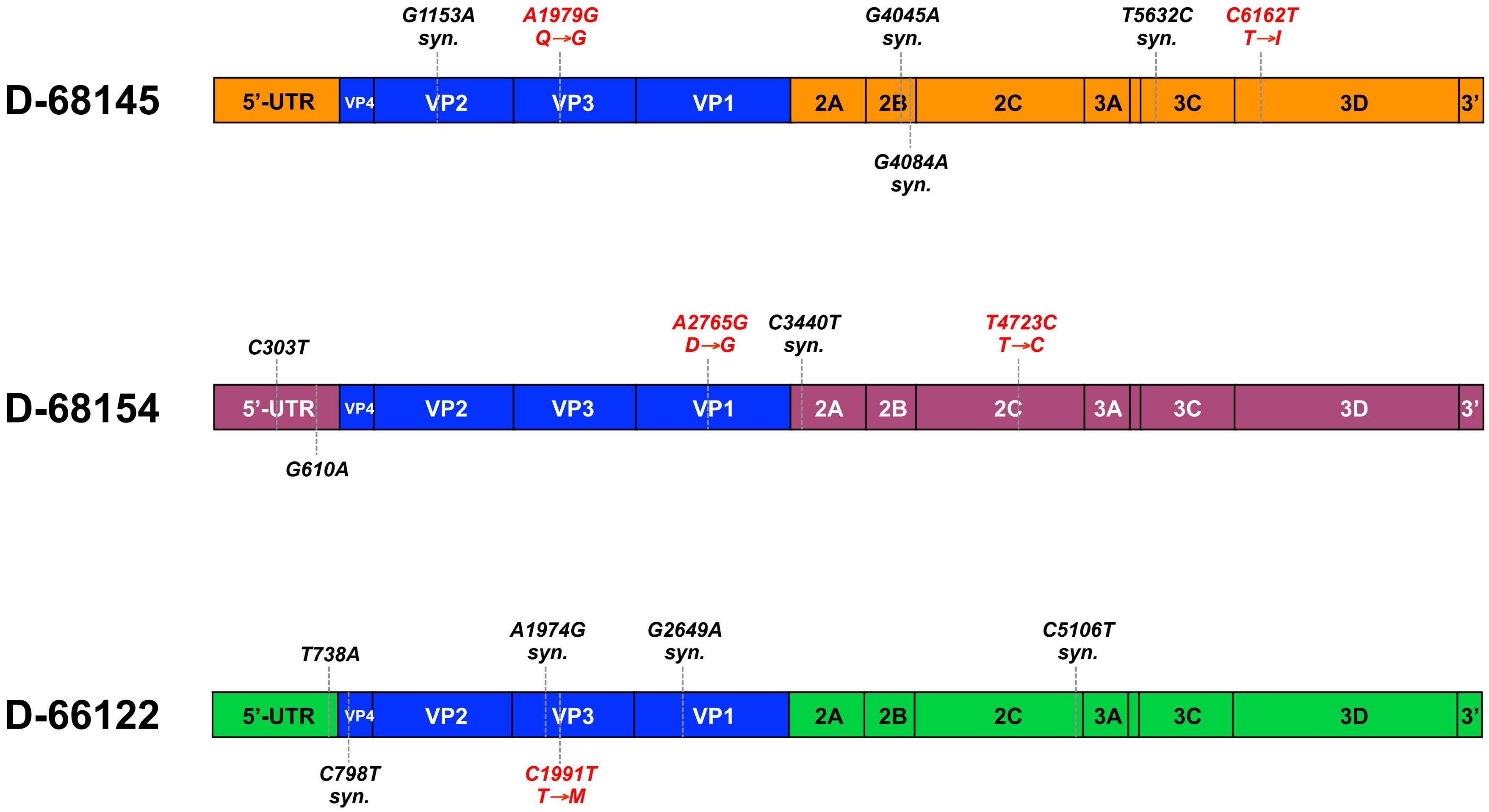

Supplementary Fig. S2. Alignment of the *cre* minimal domains (highlighted in grey) of Sabin 2 and EV-C99 68229.

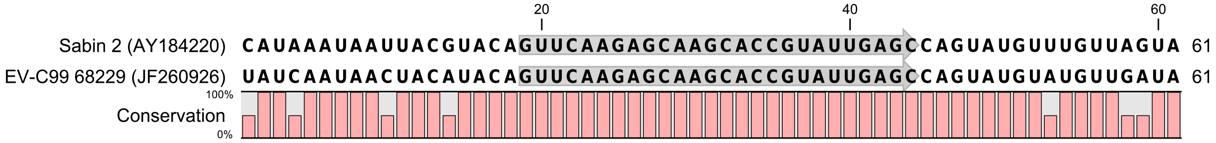

Supplementary Table S1. List of the primers used to generate the recombinant genomes.

Genome	Amplicon 1		Amplicon 2		Amplicon 3		Amplicon 4	
	Forward primer	Reverse primer	Forward primer	Reverse primer	Forward primer	Reverse primer	Forward primer	Forward primer
**F-68229**	Extr5	68229.37.c	S2.048.d	S2.022.c	68229.25.d	68229.27.c	S2.034.d	S3.002.c
**E-68229**	S2.017.d	S2.022.c	68229.25.d	68229.27.c	S2.034.d	S3.002.c		
**D-67001**	Extr5	67001.07.c	S2.038.d	S2.018.c	67001.06.d	S3.002.c		
**D-68145**	Extr5	68145.24.c	S2.047.d	S2.020.c	68145.21.d	S3.002.c		
**D-68095**	Extr5	68095.18.c	S2.037.d	S2.019.c	68095.17.d	S3.002.c		
**D-68229**	Extr5	68229.37.c	S2.048.d	S2.022.c	68229.25.d	S3.002.c		
**D-68154**	Extr5	68154.29.c	S2.036.d	S2.021.c	68154.28.d	S3.002.c		
**D-67610**	Extr5	67610.23.c	S2.049.d	S2.031.c	67610.20.d	S3.002.c		
**D-66122**	Extr5	66122.34.c	S2.050.d	S2.030.c	66122.30.d	S3.002.c		
**C-67001**	Extr5	67001.07.c	S2.038.d	S3.002.c				
**C-68145**	Extr5	68145.24.c	S2.047.d	S3.002.c				
**C-68095**	Extr5	68095.18.c	S2.037.d	S3.002.c				
**C-68229**	Extr5	68229.37.c	S2.048.d	S3.002.c				
**C-68154**	Extr5	68154.29.c	S2.036.d	S3.002.c				
**C-67610**	Extr5	67610.23.c	S2.049.d	S3.002.c				
**C-66122**	Extr5	66122.34.c	S2.050.d	S3.002.c				
**B-67001**	S2.017.d	S2.025.c	67001.08.d	S3.002.c				
**B-68145**	S2.017.d	S2.026.c	68145.22.d	S3.002.c				
**B-68095**	S2.017.d	S2.023.c	68095.19.d	S3.002.c				
**B-68229**	S2.017.d	S2.028.c	68229.26.d	S3.002.c				
**B-68154**	S2.017.d	S2.027.c	68154.30.d	S3.002.c				
**B-66122**	S2.017.d	S2.032.c	66122.31.d	S3.002.c				
**A-67001**	S2.017.d	S2.018.c	67001.06.d	S3.002.c				
**A-68145**	S2.017.d	S2.020.c	68145.21.d	S3.002.c				
**A-68095**	S2.017.d	S2.019.c	68095.17.d	S3.002.c				
**A-68229**	S2.017.d	S2.022.c	68229.25.d	S3.002.c				
**A-68154**	S2.017.d	S2.021.c	68154.28.d	S3.002.c				
**A-67610**	S2.017.d	S2.031.c	67610.20.d	S3.002.c				
**A-66122**	S2.017.d	S2.030.c	66122.30.d	S3.002.c				

Supplementary Table S2. Sequences of the primers used to generate the recombinant genomes.

Name	5'→3' sequence
66122.30.d	ATTATAAAGATGGGCTCACCCCACTACCAGAAAAGGGATTAACGACTTAT
66122.31.d	AGAGAAACAGAAGATCAAACATTGGTAATTGCATGGAAGCTCTATTCCAG
66122.34.c	TTTGAATTTTCGTGGGCTCCAACTTTCTGTGATGAAACTTGGGCGCCCAT
67001.06.d	ATTATAAAGATGGGCTCACCCCACTACCAGAAAAGGGATTAACGACTTAT
67001.07.c	TTTGAATTTTCGTGGGCTCCAACTTTCTGTGATGAAACTTGGGCGCCCAT
67001.08.d	AGAGAAACAGAAGATCAAACATTGGTAATTGCATGGAAGCTCTATTCCAG
67610.20.d	ATTATAAAGATGGGCTCACCCCACTACCAGAAAAGGGATTAACGACTTAT
67610.23.c	TTTGAATTTTCGTGGGCTCCAACTTTCTGTGATGAAACTTGGGCGCCCAT
68095.17.d	ATTATAAAGATGGGCTCACCCCACTACCAGAAAAGGGATTAACGACTTAT
68095.18.c	TTTGAATTTTCGTGGGCTCCAACTTTCTGTGATGAAACTTGGGCGCCCAT
68095.19.d	AGAGAAACAGAAGATCAAACATTGGTAATTGCATGGAAGCTCTATTCCAG
68145.21.d	ATTATAAAGATGGGCTCACCCCACTACCAGAAAAGGGATTAACGACTTAT
68145.22.d	AGAGAAACAGAAGATCAAACATTGGTAATTGCATGGAAGCTCTATTCCAG
68145.24.c	TTTGAATTTTCGTGGGCTCCAACTTTCTGTGATGAAACTTGGGCGCCCAT
68154.28.d	ATTATAAAGATGGGCTCACCCCACTACCAGAAAAGGGATTAACGACTTAT
68154.29.c	TTTGAATTTTCGTGGGCTCCAACTTTCTGTGATGAAACTTGGGCGCCCAT
68154.30.d	AGAGAAACAGAAGATCAAACATTGGTAATTGCATGGAAGCTCTATTCCAG
68229.25.d	ATTATAAAGATGGGCTCACCCCACTACCAGAAAAGGGATTAACGACTTAT
68229.26.d	AGAGAAACAGAAGATCAAACATTGGTAATTGCATGGAAGCTCTATTCCAG
68229.27.c	GGGGGACTGGTCTTAACATCTATTTTTAGATCTTTATACTGCAGTGGTCC
68229.37.c	TTTGAATTTTCGTGGGCTCCAACTTTCTGTGATGAAACTTGGGCGCCCAT
Extr5	GGGTAATACGACTCACTATAGGGTTAAAACAGCTCTGGGGTTGTTCC
S2.017.d	GGGTAATACGACTCACTATAGGGTTAAAACAGCTCTGGGGTTGTACC
S2.018.c	ACTATCTTGTACCCTGCCACATATATTGCCTTGTTTTGGTGACCAAAACC
S2.019.c	ATTATTTTATACCCTGCCACATATACGGCCTTGCTTTGATGGCCAAAGCC
S2.020.c	ACTATCTTGTACCCTGCCACATATATTGCCTTATTTTGGTGACCAAAACC
S2.021.c	CAAATTTTGAAGCCTGCAGTATACACTGCCTTGTTTTGATGTCCAAACCC
S2.022.c	ATTATTTTGTAGCCAGCAACGTATACTGCTTGATTCTGATGTCCAAATCC
S2.023.c	GGTGGACTAGTCTTGATATCTATCTTCAGGTCTTTGTACTGTATTGGTCC
S2.025.c	GGTGGGCTAGTCTTGATGTCTATCTTCAGATCCTTGTACTGTAGTGGCCC
S2.026.c	GGTGGGCTAGTCTTAATGTCTATTTTCAAATCTTTATACTGCAGCGGTCC
S2.027.c	GGGGGTGTAGTCTTTATGTCAATCTTTAGGTCTTTGTACTGCAGGGGGCC
S2.028.c	GGTGGTGTGGTCTTTATATCTATCTTCAAGTCTTTGTATTGCAATGGGCC
S2.030.c	CAAATTTTGTATCCTGCCGTGTAAACTGCTTTGTTTTGGTGTCCAAATCC
S2.031.c	CAGATTTTGAAACCTGCGGTGTATACTGCTTTGTTTTGATGTCCAAACCC
S2.032.c	GGGGGTGAGGTCTTAATATCTATTTTGAGATCTTTGTACTGGAGTGGACC
S2.034.d	AAAAGATTAGGAGAGCTAACATTGGAAATTGTATGGAAGCGCTATTCCAA
S2.036.d	CACTCCAAACCTCATTAAGATAGTGCTTTTGATACCACATCATTATCATA
S2.037.d	CACTTTGAAACTCATTGAAATTGTACTGTTGATACGGTATTACTATCACA
S2.038.d	CAGCTTGAATCTCATCAAAATTGTATTGTTGATACGACATTATTATCATT
S2.047.d	CAGCTTGAATCTCATCAAAGTTGTATTGTTGATACGACATTATTATCACA
S2.048.d	TACACCAGATTTAATTAAAGCAGTTTTGTTAATTAGATCTACATACCACA
S2.049.d	CATTGGATTCATACACTTTGCACTTGATTAGTTACGACAGCATCATCACA
S2.050.d	CACACTAAACCTAATTAGAATTACCTTGTTAATTAGACATTATTATCACA
S3.002.c	TTTTTTTTTTTTTTTTTTTTTTTTCCTCCGAATTAAAGAAAAATTTAC
